# 基于共价有机框架的色谱固定相制备及其应用研究进展

**DOI:** 10.3724/SP.J.1123.2023.04021

**Published:** 2023-10-08

**Authors:** Jin LIU, Fan WU, Lin GAN, Leyi JIN, Zian LIN

**Affiliations:** 食品安全与生物分析教育部重点实验室, 福建省食品安全分析与检测重点实验室, 福州大学化学学院, 福建 福州 350108; Ministry of Education Key Laboratory of Analytical Science for Food Safety and Biology, Fujian Provincial Key Laboratory of Analysis and Detection Technology for Food Safety, College of Chemistry, Fuzhou University, Fuzhou 350108, China

**Keywords:** 共价有机框架, 色谱固定相, 有机化合物, 异构体, 手性化合物, covalent organic frameworks (COFs), chromatographic stationary phase, organic compounds, isomers, chiral compounds

## Abstract

工业领域与科学研究的不断发展,使得复杂体系的高灵敏、高通量和高选择性分离分析面临新的挑战。色谱法在分离科学中发挥着不可替代的作用,已被广泛应用于环境监测、药物分析和食品安全等领域。由于载样量高、定量分析精确和重现性好等突出优势,基于多种保留机制的色谱分离技术已被应用于不同分析物的检测。固定相作为色谱柱的核心,对色谱的分离性能有着极其重要的影响。分离的选择性和效率极大程度上取决于所采用的固定相。然而,传统固定相如硅胶基质制备工艺复杂、pH适用范围窄,聚合物基质机械稳定性较差且易溶胀等缺点限制了其在分离领域的进一步应用。因此,开发高效的新型色谱固定相以满足不同情况下的分离要求是提高色谱分离效率的关键。共价有机框架(COFs)是一类由共价键连接而成的多孔晶体聚合物,具有低密度、高孔隙率、大比表面积和性质稳定等优点。这些突出优势使得COFs材料在分离分析领域具有潜在应用价值,且被认为是新型色谱固定相的理想材料。本文综述了最近5年基于COFs的色谱固定相的制备及其应用的最新研究进展,简要介绍了基于COFs的色谱固定相的制备,详细总结了基于COFs的固定相在色谱分离领域方面的最新应用,展望了基于COFs的色谱固定相的未来发展前景与趋势。

## Abstract (en)

Given continuous developments in industrial and scientific research, the separation and analysis of complex systems with high sensitivity, throughput, and selectivity is facing new challenges. Chromatography plays an irreplaceable role in separation science and is widely applied in environmental monitoring, pharmaceutical analysis, and food safety. Owing to their outstanding advantages, such as high loading capacity, precise quantification, and good reproducibility, chromatographic separation techniques based on various retention mechanisms have been utilized to detect different analytes. The stationary phase is the core material of chromatographic columns and has an extremely important influence on their separation performance. The selectivity and efficiency of separation largely depend on the chromatographic stationary phase. However, traditional stationary phases, such as silicon-based matrices, are characterized by complex preparation processes, poor permeability, large mass transfer resistance, and a narrow pH range. In addition, polymer matrices show poor mechanical stability and susceptibility to swelling, which limit their applications in the field of separation. Therefore, the development of novel stationary phases with the advantages of traditional stationary phases has become a research emphasis in the field of analytical science in efforts to meet separation requirements under different environments. Various stationary phases based on novel porous materials, such as metal organic frameworks (MOFs), porous organic cages (POCs), and covalent organic frameworks (COFs), are used for chromatographic separation. As mesh crystalline porous materials, MOFs have the advantages of a large surface area, adjustable structure, and easy functionalization; thus, they are widely used as chromatographic stationary phases in reverse-phase chromatography, hydrophilic-mode chromatography, mixed-mode chromatography, and other separation modes. However, because the pore size of MOFs is small and most MOFs demonstrate poor chemical stability under acidic or alkaline conditions, their applications in chromatographic separation are greatly limited. COFs are porous, crystalline polymer materials composed of light elements (H, O, C, N, B, and Si) connected via covalent bonds. Their advantages include a low density, large specific surface area, high porosity, good chemical and thermal stability, regular pores, and adjustable pore sizes. Because of their unique structures and properties, COFs are widely used in many fields such as catalysis, enrichment, gas capture, and sensing. COF materials are also suitable for separation analysis and considered ideal materials for novel chromatographic stationary phases. This review summarizes the latest research progress on the preparation and applications of COF-based chromatographic stationary phases over the past five years. First, the preparation of COF-based stationary phases (SiO_2_@COFs stationary phase, COFs monolithic stationary phase, pure COFs stationary phase and COFs-coated stationary phase) is introduced. The latest applications of COF-based stationary phases in the separation of organic compounds, isomers, and chiral compounds are then described in detail. Finally, the future development trends and challenges of chromatographic stationary phases based on COFs are discussed to provide new ideas for the future design and development of novel chromatographic stationary phases based on COFs.

生命科学、环境分析和食品安全等新兴领域的发展促使复杂体系的高分辨、高精确度和高通量分离分析不断进步。色谱是分离分析领域最重要的分离技术之一,已被广泛应用于环境监测^[1]^、药物分析^[2]^和食品安全检测^[3]^等领域。色谱分离的原理是根据待测混合物中各组分在固定相和流动相之间的分配比例不同,导致流动相洗脱过程中呈现出不同的保留时间,从而实现各组分间的分离^[4]^。色谱固定相作为色谱技术的核心,对色谱的分离性能起着极其重要的作用。常用的色谱固定相包括硅胶、氧化铝、聚合物和蛋白质等材料。硅胶和氧化铝通常被用作无机固定相,具有良好的理化性质、优异的热稳定性、较高的机械强度,可以与多种化合物进行相互作用^[5]^。聚合物则常用作有机固定相,具有较宽的pH适用范围,可以通过调节聚合度和交联度来调节其疏水性和亲水性^[6]^。蛋白质固定相则可用于生物分析和制备,可以实现对蛋白质和其他生物大分子的高效分离^[7]^。然而以上常用固定相通常也存在一些缺点,如硅胶材料的制备工艺复杂、渗透性差、传质阻力大、pH适用范围窄,聚合物材料的机械稳定性较差且易在有机溶剂中溶胀^[8]^。以上缺点在一定程度上阻碍了传统固定相的进一步发展。因此,开发兼具传统固定相优点的新型固定相以满足不同情况下的分离要求,已成为分析科学领域的研究重点之一。

目前,多种基于新型多孔材料的固定相已被用于色谱分离,如金属有机框架(metal organic frameworks, MOFs)^[9]^、多孔有机笼(porous organic cages, POCs)^[10]^和共价有机框架(covalent organic frameworks, COFs)^[11]^等。MOFs作为一种网状结晶多孔材料,具有比表面积大、结构可调和易于功能化等优点^[12]^,目前已被广泛报道作为色谱固定相应用于反相色谱^[13]^、亲水模式色谱^[14]^和混合模式色谱^[15]^等多种分离模式中。然而MOFs的孔径尺寸较小(<2 nm)且多数MOFs在酸性或碱性条件下化学稳定性较差,这极大地限制了其在色谱领域中的应用。共价有机框架是一类由轻质元素(H、O、C、N、B、Si)通过共价键连接而成的多孔晶体聚合物材料,具有密度低、比表面积大、性质稳定、孔道规整、孔径可调等优点^[16,17]^。由于其独特的结构和性质,COFs被广泛应用于催化^[18]^、富集^[19,20]^、气体捕获^[21]^、样品前处理^[22,23]^和传感^[24]^等多个领域,并被认为是新型色谱固定相的理想材料。

本文针对基于COFs的色谱固定相制备及其应用的最新研究进展进行了全面总结。表1列出了近5年来开发的典型固定相,包括合成方法、目标分析物、分离模式或方法^[11,25⇓⇓⇓⇓⇓⇓⇓⇓⇓⇓⇓⇓⇓⇓⇓⇓⇓⇓⇓-45]^。本文首先介绍了基于COFs的色谱固定相的制备(SiO_2_@COFs色谱固定相、纯COFs色谱固定相和COFs涂层色谱固定相),然后详细介绍了基于COFs的固定相在有机化合物分离、异构体分离和手性化合物分离中的最新应用。最后讨论了基于COFs的色谱固定相的未来发展趋势与挑战,为进一步设计和开发基于COFs的新型色谱固定相提供了新的思路。

**表 1:** 近5年开发的典型固定相、合成方法、目标分析物和分离模式/方法

Stationary phases	Synthesis methods	Target analytes	Separation modes/methods	Ref.
COFs	quiescence in room- temperature synthesis	anilines, alkylbenzenes, halogenated nitrobenzenes, phthalates	RP-HPLC	[11]
SiO_2_@COF	one-pot synthesis	alkylbenzenes, PAHs, positional isomers, nucleosides, aniline, sulfanilamide	RP-HPLC/HILIC	[25]
CCOF 17 and 18	solvothermal synthesis	racemates (amino acids, esters, lactones, amides, alcohols, aldehydes, ketones, drugs)	GC/HPLC	[26]
NPS@TPB-DMTP	in-situ polymerization	monosubstituted benzenes, PAHs, alkylbenzenes, anilines, phthalates	RP-HPLC	[27]
S_F-COFs_	quiescence in room- temperature synthesis	alkylbenzenes, PAHs, aromatic amines, perfluoroalkyl methacrylates, halogenated trifluorotoluenes, polyfluorobenzenes, polychlorobenzenes, polybromobenzenes	RP-HPLC	[28]
COF-300	quiescence in room- temperature synthesis	alkylbenzenes, monosubstituted aromatics, PAHs, the mixture of ethylbenzene, positional isomers (nitroaniline, dihalogenated benzene, diethylbenzene, halogenated nitrobenzene)	RP-HPLC	[29]
3D-IL-COF-1	solvothermal synthesis	alkylbenzenes, PAHs, acidic compounds, basic compounds, isomers	RP-HPLC	[30]
Lysozyme CCOF 1	solvothermal synthesis	racemates (DL-threonine, DL-leucine, DL-tryptophan, ofloxacin, metoprolol, chlorpheniramine)	NP/RP-HPLC	[31]
CCOF 5 and 6	bottom-up synthesis	racemic alcohols (1-phenyl-2-propanol, 1-phenyl-1-pentanol, 1-phenyl-1-propanol, 1-(4-bromophenyl)ethanol)	NP-HPLC	[32]
CTpBD@SiO_2_	in-situ growth synthesis	racemates (alcohols, bases, phenols, ketones, organic acids, amines)	NP-HPLC	[33]
Sil-COF-CD	room-temperature solvothermal synthesis	alkylbenzenes, PAHs, isomer (o-terphenyl, m-terphenyl, p-terphe- nyl, triphenylene), 2-phenylpropionic acid, 1-phenyl-1-propanol	RP-HPLC	[34]
CDMPC@SCOF	quiescence in room- temperature synthesis	racemates (metalaxyl, 1-(1-naphthalenyl)ethanol, epoxiconazol, trans-stilbene oxide)	NP-HPLC	[35]
COF CTzDa	quiescence in room- temperature synthesis	amino acids (tryptophan, serine, cysteine, aspartic acid, histidine)	RP-HPLC	[36]
β-CD-COF	room-temperature stirring synthesis	linear alkanes, linear alcohols, fatty acid methyl esters mixture, the Grob mixture, positional isomers, chiral compounds (chiral alcohols, aldehydes, ethers, amino acid derivatives)	GC	[37]
CTzDva	solvothermal synthesis	benzene/cyclohexane, racemates (citronellal and fenchone)	GC	[38]
TzDHNDA-PEAA	solvothermal synthesis	isomers (fluoroaniline, chloroaniline, nitrotoluene, pinene, ionone, 1,3-dichloropropene)	GC	[39]
JNU-5	building-block exchange synthesis	isomers (xylene, dichlorobenzene, propylbenzene)	GC	[40]
TFPB-BD	in situ room-temperature synthesis	neutral compounds, phenols, anilines, amino acids, parabens	CEC	[41]
TpTAM	ultrasound-assisted synthesis	fluoroquinolones (enroflfloxacin, danoflfloxacin mesylate, saraflfloxacin hydrochloride, ciproflfloxacin hydrochloride)	CEC	[42]
β-CD COF_BPDA_	quiescence in room- temperature synthesis	β-blockers (timolol, propranolol, sotalol, atenolol, metoprolol), β-agonist (salmeterol), antihistamines (promethazine, chlorphenamine), tropane alkaloid (homatropine)	CEC	[43]
TAPB-BPTA	in-situ growth synthesis	alkylbenzenes, phenols, chlorobenzenes, non-steroidal anti-inflammatory drugs, parabens	CEC	[44]
COF-V	in-situ growth synthesis	neutral compounds, phenols, antiepileptic drug, triazine herbicides, active ingredients of Chinese medicine	CEC	[45]

PAHs: polycyclic aromatic hydrocarbons; HILIC: hydrophilic interaction liquid chromatography; NP: normal phase.

## 1 共价有机框架色谱固定相的制备

### 1.1 SiO_2_@COFs色谱固定相

COFs孔道规整、比表面积大和性质稳定等优异性能使其在色谱分离领域具有良好的应用前景。然而,目前报道的大部分COFs存在形貌不规则,粒径分布宽和具有纳米尺寸等特点,使得将其直接作为色谱固定相成为挑战。为解决这一关键问题,研究者们通过将COFs与形貌规则的基质相结合来作为色谱固定相。

将COFs与球形SiO_2_基质结合制备核-壳结构固定相是开发新型高效液相色谱(HPLC)固定相的常见方法之一。Zheng等^[25]^以均苯四甲酸二酐和1,3,5-三-(4-氨基苯基)三嗪为单体,通过“一锅法”合成了条形COF,并通过溶剂热法将其键合到SiO_2_表面,用于HPLC分离。通过观察流动相对保留时间的影响,研究了SiO_2_@COF的保留机理。结果表明,所制备的固定相具有疏水、亲水、氢键和*π-π*相互作用等多种相互作用模式,可以实现极性和非极性分析物的高效分离。Liu等^[46]^以三醛基间苯三酚(Tp)与2-硝基-1,4-苯二胺(Pa-NO_2_)为单体合成了TpPa-NO_2_,并对其进行还原,再通过后修饰策略将D-葡萄糖修饰到还原后的TpPa-NH_2_上,制备得到TpPa-NH_2_-Glu。随后将其固载到球形硅胶表面,用于HPLC分离。所制备的固定相具有良好的分离效果,在反相(RP)/正相(NP)模式下,成功实现了外消旋体化合物和苯系位置异构体的拆分。Yuan等^[26]^合成了两个具有手性冠醚基团的烯烃连接COFs,并采用“网包法”将其固载到SiO_2_上(图1)。所获得的两种手性固定相具有较高的稳定性,可同时应用于GC和HPLC的分离中。手性冠醚在COFs通道内呈现周期性排列,允许通过分子间相互作用对客体分子进行对映选择性识别,从而实现多种外消旋化合物的分离。此外,Xie等^[27]^以无孔二氧化硅球(NPS)为载体,COFs TPB-DMTP(TPB: 1,3,5-三(4-氨基苯基)苯; DMTP: 2,5-二甲氧基-1,4-苯二醛)为多孔功能壳,通过在NPS表面进行原位聚合,制备得到NPS@TPB-DMTP固定相用于HPLC分离,成功实现了一系列小分子化合物的快速基线分离。在这一固定相中,该填料的分离仅发生在表面多孔层,NPS芯仅作为支撑载体,从而消除了硅胶孔隙的影响,保证了COFs功能层在分离过程中的独立性。这为利用COFs优异的分子水平可设计性优势,在介观尺度调控固定相的结构和功能提供了可能。

**图 1:**
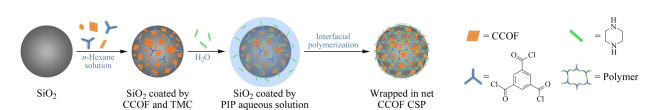
“网包法”制备COFs手性固定相的示意图^[26]^

### 1.2 COFs整体色谱固定相

除了将COFs与球形SiO_2_基质结合制备色谱固定相外,将可渗透多孔单体与COF(COF-单体)结合作为固定相原位聚合于色谱柱内,同样有望解决COFs在HPLC中柱压力高和效率低等问题。Qian等^[36]^以D-樟脑酸功能化4,4',4'-(1,3,5-三嗪-2,4,6-三基)三苯胺(CTz)和1,4-二羟基对苯二甲醛(Da)为配体制备得到手性COF CTzDa,并将其掺入乙烯二甲基丙烯酸甲酯和甲基丙烯酸酯基质的可渗透单体中,形成CTzDa单体。通过系统的实验表征、热动力学分析和分子对接计算,验证了CTzDa单体的良好渗透性和机械稳定性,随后将其用于氨基酸(AAs)的分离。CTzDa和AAs之间存在的立体氢、*π-π*和范德华相互作用以及手性COFs易于结合到多孔单体中,促进了手性CTzDa对AAs的选择性分离。2021年,Liu等^[30]^报道了首个用于HPLC的三维COFs集成整体柱。其3D-IL-COF-1整体柱材料以四(4-甲酰苯基)-甲烷和对苯二胺为原料,采用典型的溶剂热法制备而成,具有纳米多孔5倍互穿介质网络结构。随后将该材料掺入甲基丙烯酸-二甲基丙烯酸乙酯单体中,得到3D-IL-COF-1整体柱。该方法优于已报道的离子热合成方法,得到的3D-IL-COF-1整体柱结构均匀,渗透性好,力学稳定性高。多孔有机整体基质保证了制备的COFs整体柱的渗透性,3D-IL-COF-1的引入为整体柱提供了多种相互作用,促进了其对中性、酸性和碱性化合物的高效分离。

### 1.3 纯COFs色谱固定相

2019年,我们课题组在室温下可控合成了从纳米到微米尺度的均匀球形COFs^[47]^。得到的不同粒径的球形COFs具有超高的表面积、良好的结晶度和化学/热稳定性,这使得选择纯COFs直接作为HPLC固定相成为可能。基于以上球形COFs的制备基础,我们尝试将单分散球形COFs直接填入色谱柱作为色谱固定相^[11]^。实验以1,3,5-三(4-氨基苯基)苯和2,5-二乙烯基对苯二醛为原料,乙酸溶液为催化剂,室温下可控合成微米级球形COFs(S_COFs_)用于HPLC分离(图2a)。结果表明,该S_COFs_填充柱不仅在疏水性小分子的分离中获得了高柱效和良好的分离度,还成功应用于复杂生物样品(牛血清白蛋白酶解液)的分离。2022年,我们组合成了氟功能化球形COFs(S_F-COFs_),并将其作为色谱固定相用于有机卤化物的分离^[28]^。得益于S_F-COFs_良好的单分散性、疏水性和高含氟量,S_F-COFs_填充柱成功实现了有机卤化物的分离。值得注意的是,在相同条件下,不含氟COFs填充柱无法对以上有机卤化物进行分离,这揭示了氟元素的引入对分离效果产生了重要影响。基于以上工作,我们继续探索具有规则形貌的非球形COFs作为HPLC固定相的分离效果,制备了单晶COFs(COF-300)固定相用于有机小分子和位置异构体的分离(图2b)^[29]^。该COF-300固定相具有规则的形状、微孔特性以及优异的化学和热稳定性,不仅可以用于疏水性小分子的分离,还可以实现物理化学性质相近的位置异构体的分离。同时,与多晶COF-300填充柱和商品化色谱柱进行分离性能的对比,进一步证实了单晶COF-300固定相的优异分离性能。以上工作不仅验证了将具有规整形貌的纯COFs直接作为液相色谱固定相的可行性,同时也进一步拓宽了COFs在色谱分离中的应用。

**图 2:**
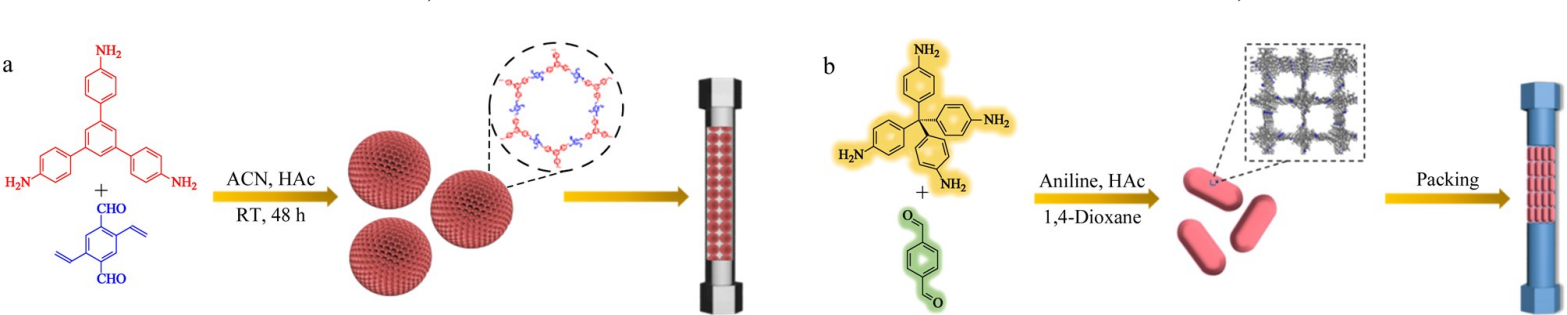
(a)单分散S_COFs_固定相^[11]^和(b)单晶COF-300固定相的制备示意图^[29]^

### 1.4 COFs涂层色谱固定相

COFs涂层作为色谱固定相具有高度可调性、高选择性和高灵敏性等特点。Fu等^[41]^采用1,3,5-三-(4-甲酰苯基)苯(TFPB)和联苯胺(BD)作为有机单体,合成了COF (TFPB-BD),并将其引入到醛基修饰的毛细管中,室温下原位合成COF(TFPB-BD)涂层固定相。所制备的固定相具有较高的比表面积、足够的有效相互作用位点和良好的传质性能,成功实现了有机化合物的毛细管电色谱(CEC)分离(图3a)。Zong等^[42]^首次采用超声辅助法合成了三维COFs TpTAM。随后,将其键合到毛细管柱中,并应用于氟喹诺酮类药物的分离(图3b)。结果表明,所制备的TpTAM涂层毛细管柱具有良好的稳定性,重复使用100次后分离效率仍未有明显变化。随后对可能存在的分离机理进行探讨,发现*π-π*堆积效应、疏水相互作用和氢键作用是影响分离的主要因素。

**图 3:**
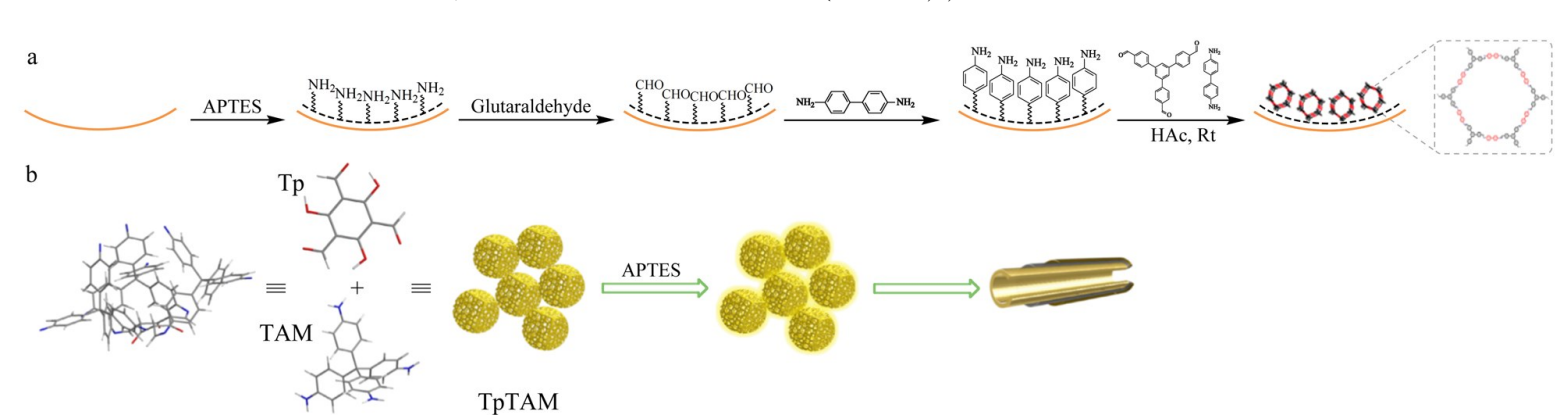
(a)TFPB-BD@毛细管柱^[41]^和(b)TpTAM涂层毛细管柱的制备示意图^[42]^

*β*-环糊精(*β*-CD)及其衍生物是一类具有手性中心的有机化合物,被广泛应用于分子的特异性识别。Wang等^[43]^通过光聚合法,将4,4'-联苯二甲醛和七(6-氨基-6-脱氧)-*β*-环糊精(Am7CD)缩合而成的手性COF(*β*-CD COF_BPDA_)引入到毛细管色谱柱中。*β*-CD COF_BPDA_具有较大的孔径和比表面积,此外*β*-CD COF_BPDA_的包被使手性固定相具有优越的三维取向,从而对十几种外消旋药物表现出了满意的分离效果。2021年,Tang等^[37]^以Am7CD和对苯二甲醛为原料制备得到*β*-CD-COF,并将其涂覆于毛细管内表面制备得到手性*β*-CD-COF包覆的毛细管色谱柱。得益于*β*-CD手性单元的整合,该固定相不仅存在永久的手性空间,同时还具备较高的热稳定性和较大的表面积。同年,Guo等^[38]^选择具有C=C键反应位点的2,5-二乙烯基-1,4-苯甲醛为单体,与2,4,6-三(4-氨基苯基)-1,3,5-三嗪缩合,合成乙烯基功能化COFs。随后,又合成了手性硫衍生物(*R*)*/*(*S*)*-n*-(2-巯基乙基)-2-苯基丙酰胺,并通过“巯基”点击反应将其与TzDva结合,制备得到具有较高热稳定性和良好孔隙率的手性COF CtzDva。随后将CtzDva作为气相色谱固定相,实现了外消旋化合物的高效分离。以上研究不仅为COFs的合成提供了新的前景,同时也扩展了COFs在分离领域的进一步应用。

## 2 共价有机框架色谱固定相的应用

### 2.1 有机化合物的分离

色谱技术是分离分析的重要工具之一,具有高效、精确、可靠的优点。目前,基于COFs的色谱固定相已被广泛应用于有机化合物的广谱分离。Liu等^[30]^采用溶剂热法制备得到三维COFs固定相3D-IL-COF-1,并将其纳入整体柱中。结果表明,该整体柱不仅可用于中性化合物烷基苯(苯、甲苯、乙苯、丙烯和丁基苯)和多环芳烃(苯、萘、蒽、芘和苯并芘)的基线分离,同时也对酸性苯酚类化合物(间硝基苯酚、2,6-二甲基苯酚和2,6-二氯苯酚)和碱性苯胺类化合物(苯胺、间硝基苯胺和*N*,*N*-二甲基苯胺)具有良好的分离效果(图4)。采用乙腈-水(70/30, v/v)为流动相,在流速为0.2~2 mL/min的范围内,3D-IL-COF-1整体柱上甲苯的最高理论塔板数为20700 plates/m,显著高于空白整体柱(9541 plates/m)。He等^[44]^采用室温原位生长法制备了COF TAPB-BPTA(1,3,5-三(4-氨基苯基)苯(TAPB), 2,5-双(2-丙炔-1-基氧基)-1,4-苯二甲醛(BPTA))改性毛细管柱。所制备的色谱柱不仅对烷基苯、酚类和氯苯类化合物表现出优异的分离性能,还在非甾体抗炎药和对羟基苯甲酸酯的基线分离中具有良好的效率和分辨率,同时,在日内(*n*=5)、日间(*n*=3)和平行柱(*n*=3)中均具有较好的精密度,所测烷基苯保留时间的相对标准偏差均小于2.58%。以上结果表明,基于COFs的新型固定相在有机化合物的分离领域具有巨大的应用潜力。

**图 4:**
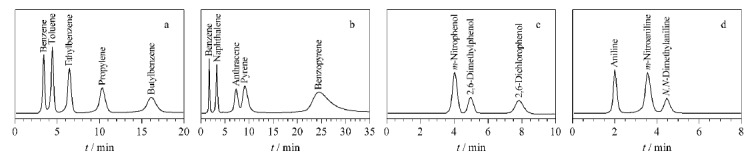
中性、酸性和碱性化合物在3D-IL-COF-1整体柱上的高效液相色谱图^[30]^

### 2.2 异构体的分离

异构体在工业领域被广泛使用,由于具有相似的物理化学性质,异构体的分离一直是分离领域的重点和难点。虽然蒸馏技术已被用于异构体的分离,但这种热驱动过程不仅耗时且效率较低^[48]^。近年来,基于COFs的色谱固定相逐渐被开发出来用于异构体的分离。Ma等^[39]^采用后修饰策略制备得到了[(1-苯乙基)氨基]乙酸(PEAA)功能化的COFs(COF TzDHNDA-PEAA),随即将其涂覆于毛细管柱中作为GC固定相。该柱不仅可以对正十二烷进行高效分离(12417 plates/m, 120 ℃),同时对氟苯胺(*R_m-/o_*_-_=3.2, *R_p-/m_*_-_=2.4)、氯苯胺(*R_m-/o_*_-_=5.7, *R_p-/m_*_-_=1.8)、硝基甲苯(*R_p-/o_*_-_=2.2, *R_m-/p_*_-_=1.7)、紫罗酮(*R_β-/α_*_-_=4.8)、蒎烯(*R_β-/α_*_-_=2.4)和1,3-二氯丙烯(*R_E-/Z_*_-_=2.0)等多种异构体也表现出了较高的分辨率(*R*_s_)。PEAA的引入丰富了涂层中苯环、仲胺和羰基的含量,增强了氢键和*π-π*相互作用,从而实现了异构体的高分辨率分离。Qian等^[40]^采用构建块交换策略合成了具有丰富孔道和笼孔的氨基三维共价有机框架(JNU-5)固定相,并用于异构体的分离。在JNU-5-毛细管柱上,二甲苯、二氯苯和丙基苯3种异构体在3.5 min恒温条件下均获得了良好的基线分离(*R*_s_=1.85~2.89)和高柱效率(间二氯苯为11429 plates/m)。与商用毛细管色谱柱(DB-5毛细管柱和HP-FFAP毛细管柱)和已报道的新型材料基毛细管色谱柱(微孔有机聚合物、石墨烯量子点、2D COF BtaMth、MOF-CJ3、沸石金属偶氮酸盐框架MAF-6和MOF UiO-66)相比,JUN-5-毛细管色谱柱在分离芳香异构体,特别是二甲苯异构体时表现出更好的分辨率。我们组采用室温法制备了单晶COF-300固定相,用于位置异构体的分离^[29]^。如图5所示,该COF-300填充柱实现了硝基苯胺、二氯苯、二溴苯、二碘苯、二乙苯、氯硝基苯、溴硝基苯和碘硝基苯8种位置异构体的高分辨率分离。*m-/p*-二碘苯和*o-/m*-二碘苯的分辨值分别为4.45和2.53。此外,将COF-300填充柱与商业高碳柱和C18柱进行了分离性能的对比,可以看出单晶COF-300固定相对于位置异构体的分离具有显著优势。

**图 5:**
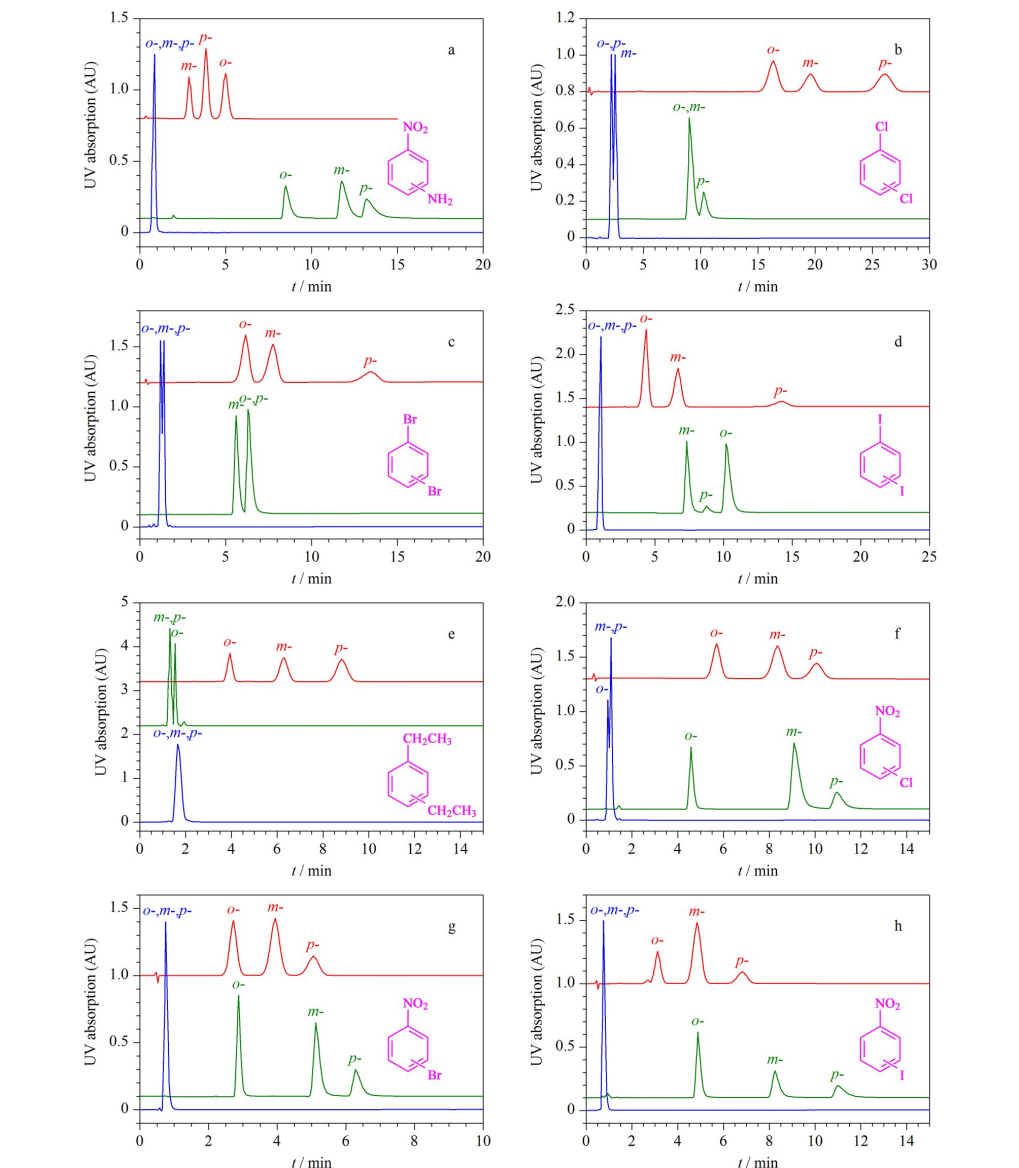
在单晶COF-300填充柱(红色)、商业高碳柱(绿色)和C18柱(蓝色)上分离位置异构体^[29]^

### 2.3 手性化合物的分离

手性化合物是一类非对称分子,其分子镜像之间不能通过旋转或平移相互重合,这类分子结构的不对称性使得它们具有非常重要的药理学和生物学意义。因此分离和纯化手性化合物是化学和药学等领域的关键问题之一^[49]^。2018年,Zhang等^[31]^首次将一系列生物分子(溶菌酶、三肽和L-赖氨酸)锚定于非手性COFs中。所获得的生物分子COFs保留了生物醇的强手性和特异性,在NP和RP HPLC中均表现出对各种消旋体(药物和AAs等)的高手性分离效率。同年,Han等^[32]^报道了由四面体四胺和手性四醛通过亚胺缩合反应制备得到的三维手性COFs(CCOF 5),随后通过对亚胺键进行氧化,将骨架转化为酰胺键COFs(CCOF 6)。所合成的两种COFs具有4倍互穿的金刚烷类开放框架,管状通道装饰有手性二羟基助剂。在最优条件下,CCOF 5填料柱在40 min内获得了良好的分离因子(*α*=1.19)和色谱分辨率(*R_s_*=1.52)。CCOF 6填充柱可以实现1-苯-2-丙醇、1-苯基-1-戊醇、1-苯-1-丙醇和1-(4-溴苯基)-乙醇等外消旋体的基线分离,*α*/*R*_s_分别为1.29/1.78、1.21/1.58、1.33/2.47和1.24/1.54。微孔COFs在小分子的分离中具有巨大潜力,但其设计与合成仍存在困难。Huang等^[50]^采用乙二胺与四面体四(水杨醛)硅烷/甲烷衍生物的席夫碱反应制备了两对微孔三维salen基和Zn(salen)基COFs。所制备的COFs具有7倍互穿菱形开放框架,并表现出永久孔隙。将非配位salen基团功能化的两种COFs作为HPLC的固定相,成功获得了对二甲苯(px)、间二甲苯(mx)、邻二甲苯(ox)和乙苯(EB)的基线分离以及良好的分离因子(*α*_px/Eb_=1.4, *α*_mx/px_=1.3, *α*_ox/mx_=2.0)与分辨率(*R*_px/Eb_=1.7, *R*_mx/px_=1.5, *R*_ox/mx_=5.2)。此外,Guo等^[33]^以三醛基间苯三酚、(+)-二乙酰-L-酒石酸酐和联苯胺为原料,制备得到手性COF CTpBD。随后采用原位生长法将其固定在氨基功能化二氧化硅表面,制备手性COF核壳微球复合物CTpBD@SiO_2_,并应用于醇类、碱类、酚类、酮类、有机酸类和胺类等外消旋体的分离。结果表明,所制备的CTpBD@SiO_2_填充柱具有较高的柱效率(阿替洛尔为16800 plates/m),最高*R*_s_为2.11, *α*为3.73。Wan等^[34]^利用室温溶剂热法制备均匀球形COF改性二氧化硅(Sil-COF),然后利用硫烯点击化学将巯基化*β*-环糊精修饰到Sil-COF上。制备得到的Sil-COF-CD固定相不仅成功分离了3种非极性物质(烷基苯、多环芳烃和异构体),同时也实现了手性对映体(2-苯基丙酸和1-苯基-1-丙醇)的基线分离。此外,与商品*β*-CD色谱柱相比,Sil-COF-CD柱展现出更强的手性识别能力,进一步证实了其在手性化合物分离中的优势(图6)。以上研究不仅促进了COFs在手性分离中的应用,同时为设计高效、耐用的新型手性固定相提供了有价值的指导。

**图 6:**
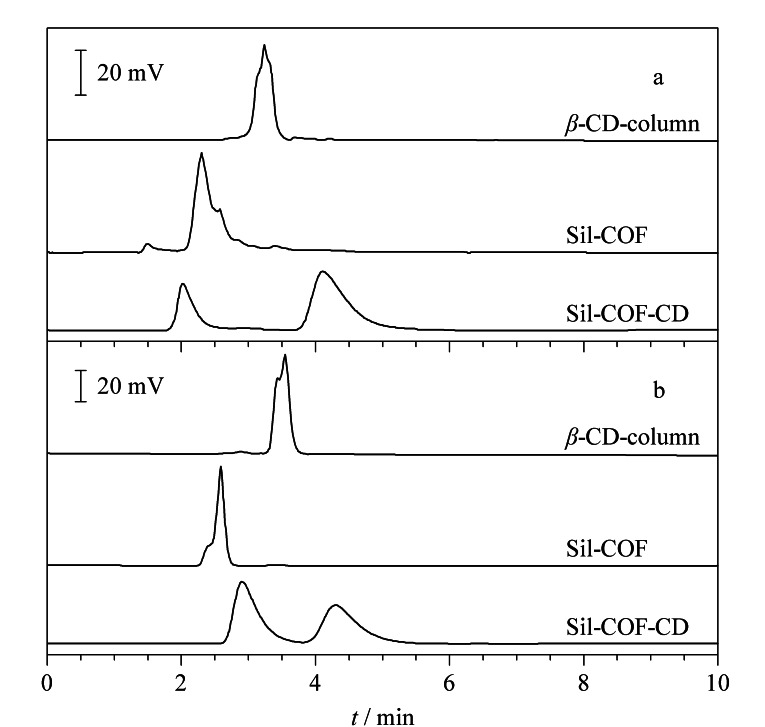
手性对映异构体(a)2-苯基丙酸和(b)1-苯基-1- 丙醇在Sil-COF柱、Sil-COF-CD柱和商用*β*-CD柱上的色谱分离图^[34]^

## 3 总结与展望

综上所述,我们总结了基于COFs的色谱固定相的制备及其在色谱分离领域中的应用。介绍了SiO_2_@COFs色谱固定相、COFs整体色谱固定相、纯COFs色谱固定相和COFs涂层色谱固定相的制备,并综述了基于COFs的固定相在有机化合物的广谱分离化合物以及异构体和手性化合物分离中的应用。COFs作为一类新型多孔材料,其固有的优势包括低密度、大的比表面积、良好的热和化学稳定性、可调的孔径等使其在色谱分离领域展现出了良好的应用潜能。目前,越来越多基于COFs的色谱固定相被相继开发出来用于提高对待测物的分析灵敏度和选择性。然而现有的COFs合成方法仍存在合成条件苛刻、制备时间长、成本高昂等缺点,在一定程度上限制了其在色谱分离领域中的应用。在手性COFs的制备方面,平衡COFs晶体结构的对称性和手性结构的不对称性仍然是研究的难点。这意味着在未来COFs的研究中,需要探索新的手性COFs合成方法如微波辅助法、室温合成和化学合成法等,同时可以考虑通过除亚胺键外的其他键,如烯烃、吩嗪、酰亚胺等化学键来对COFs进行连接。此外,为了提高COFs的分离性能,还可以对其进行特定的设计和功能化。未来,也可以通过将计算模拟和实验研究相结合,进一步优化COFs的结构和性能,实现对更多化合物的选择性吸附和分离。最后,色谱固定相分离机理的研究仍然不够深入,如手性COFs分离外消旋体的机理尚不明确,还亟待研究。因此,有必要进一步对COFs的分离机理进行探究,从而为设计具有更好分离效果的COFs提供理论指导。随着具有优良性能的新型COFs不断涌现,相信COFs将在色谱固定相的研究中获得飞速发展,并在色谱分离领域中收获更广阔的应用前景。
